# Cucurbitacin IIa: a novel class of anti-cancer drug inducing non-reversible actin aggregation and inhibiting survivin independent of JAK2/STAT3 phosphorylation

**DOI:** 10.1038/bjc.2011.10

**Published:** 2011-02-08

**Authors:** C Boykin, G Zhang, Y-H Chen, R-W Zhang, X-E Fan, W-M Yang, Q Lu

**Affiliations:** 1 Department of Anatomy and Cell Biology, East Carolina University, Greenville, NC 27834, USA; 2 Leo Jenkins Cancer Center, The Brody School of Medicine, East Carolina University, Greenville, NC 27834, USA; 3 Longjin Pharmaceuticals, Kunming, Yunnan 650228, China; 4 Yunnan Key Laboratory of Pharmacology for Natural Products, Kunming Medical University, Kunming, Yunnan 650031, China

**Keywords:** cucurbitacin IIa, RhoA, survivin, JAK2/STAT3, actin cytoskeleton, apoptosis

## Abstract

**Background:**

Cucurbitacin (Cuc) and triterpene-derived natural products exhibit anti-cancer potential in addition to their conspicuous anti-bacterial and anti-inflammatory activity. Recently, inhibition of Janus kinase 2 (JAK2)/signal transducer and activator of transcription 3 (STAT3) signaling was shown to underlie the effects of Cuc family on inducing cell death in cancer.

**Method:**

We purified Cuc IIa, the active component from the medicinal plant *Hemsleya amalil*s Diels, which shows different structural modifications from other Cuc derivatives. We investigated the mechanisms of its inhibitory effects on cancer cells *in vitro* and tumour growth *in vivo*.

**Results:**

Cuc IIa induced the irreversible clustering of filamentous actin and arrested cell cycle by the increases in G2/M populations. Cuc IIa resulted in the reduced phospho-Histone H3 and markedly increased cleavage of poly-(ADP-ribose) polymerase or PARP, immediate upstream of DNA breakdown as the result of caspase activation, consistent with mitotic blockage-induced cell death. However, unlike other Cuc members, Cuc IIa did not suppress JAK2/STAT3 phosphorylation or alter phosphorylation of mitogen-activated protein kinases. Instead, the expression of the cell cycle-regulated Inhibitor of Apoptosis Protein (IAP) survivin was reduced. Introducing oncoprotein *δ*-catenin, which increased survivin expression and suppressed small GTPase RhoA, reduced efficacy of Cuc IIa to induce cell death. Supporting the effects of Cuc IIa on actin cytoskeletal signaling, RhoA phosphorylation was reduced suggesting its increased activity.

**Conclusion:**

Cuc IIa is a novel class of anti-cancer drug in suppression of cancer cell expansion by disrupting the actin cytoskeleton and directing the cell to undergo PARP-mediated apoptosis through the inhibition of survivin downstream of JAK2/STAT3.

Cancer development is the result of loss of balance in cellular functions including cell growth, adhesion, division, and apoptosis (Cotter, 2009). A number of signal transduction pathways are involved in the alteration of cell functions in cancer (Manning and Cantley, 2007; Karnoub and Weinberg, 2008). To develop rationally designed therapies for cancer treatment, intensive researches aimed at the identification of novel drugs to inhibit cancer cell-specific signaling or restore cell signaling to the normal states.

Triterpene-derived natural products from medicinal plants exhibit conspicuous anti-bacterial and anti-inflammatory effects. In the past several years, cucurbitacins have become a subject of intense investigation because this triterpene family induces cancer cell death (Wu *et al*, 2002; Sun *et al*, 2005; Liby *et al*, 2007) and many of them inhibit the phosphorylation of Janus kinase 2 (JAK2) and signal transducer and activator of transcription 3 (STAT3) (Bromberg *et al*, 1999; Blaskovich *et al*, 2003; Sun *et al*, 2005; Thoennissen *et al*, 2009). STAT3, originally identified as an important regulator of lymphocyte interferon-mediated gene transcription, is constitutively activated in many cancers (Bromberg *et al*, 1999). Although JAK2/STAT3 signaling can conceivably be controlled at the levels of growth factor ligands, surface receptors and intracellular downstream target elements, it is previously reported that cucurbitacins shut down JAK2/STAT3 signaling by inhibiting their phosphorylation.

Cucurbitacin IIa (Cuc IIa) is a triterpene family component of natural products with different structural modifications from other Cuc derivatives. This chemical can be isolated from the *Hemsleya amalils* Diels plant. The plant has been used as a homoeopathic treatment for various diseases in China for centuries and has been indicated to display anti-cancer potential (Wu *et al*, 2002). However, the molecular and cellular mechanisms of its anti-cancer activities have not been established.

In this study, we demonstrate that Cuc IIa inhibits cancer cell expansion by disrupting the actin cytoskeleton and mitosis. However, unlike other Cuc derivatives which induce cell death through the inhibition of JAK2/STAT3 signaling, Cuc IIa directs the cells to undergo apoptosis independent of JAK2/STAT3 phosphorylation but targets their potential downstream component Inhibitor of Apoptosis Protein (IAP)/survivin and poly-(ADP-ribose) polymerase (PARP) pathways. Therefore, actin cytoskeletal signaling is the common target of Cuc family's anti-cancer activity, which can converge on cell survival machinery through JAK2/STAT3-dependent and -independent mechanisms.

## Materials and methods

### Antibodies and reagents

Antibodies against total PARP, cleaved PARP, Histone H3, STAT3, phospho-STAT3 (tyrosine 705), JAK2, phospho-JAK2 (tyrosine 1007/1008), ERK1/2, and phospho-ERK1/2, were from Cell Signaling (Danvers, MA, USA). Rabbit anti-phosphorylated RhoA (serine 188), sepharose beads conjugated anti-RhoA, and sepharose beads conjugated anti-Cdc42 were from Santa Cruz Biotech (Santa Cruz, CA, USA). Anti-survivin, phospho-Histone H3, and phosphorylated Rac1/Cdc42 (serine 71) were obtained from Millipore (Billerica, MA, USA). Monoclonal anti-tubulin (DM1*α*) was from Sigma (St Louis, MO, USA). Monoclonal anti-actin (JL20) and anti-GAPDH were from EMD Science (Gibbstown, NJ, USA). All other chemicals were from Sigma unless indicated otherwise.

### Extraction and isolation of Cuc IIa from Hemsleya

Dried and finely powdered roots of Xuedan (*H. amalils* Diell) were extracted with acetone at room temperature for 10 days and filtrated. The extracts were concentrated in water bath and subjected to chromatography on a silica gel column. The crude Cuc IIa was eluted with chloroform containing increasing volume of methanol (10–20%). After further purification by ethanol recrystallisation, Cuc IIa was obtained with over 98% purity, as determined by HPLC. The structure of Cuc IIa was confirmed by UV, IR, NMR, and MS analysis (Zhang *et al*, 2006).

### Cell culture

The mouse hepatocellular carcinoma H22, human lung cancer NCI-H1299, human prostate cancer PC-3, and CWR22Rv-1 were grown in RPMI 1640 with 10% fetal bovine serum (FBS). The mouse NIH3T3 cells were grown in Dulbecco's modified Eagle's medium (DMEM) with 10% FBS (Gibco, Carlsbad, CA, USA). Lewis lung carcinoma (LLC) cell lines were maintained in DMEM. All cells were incubated at 37°C in 5% CO_2_ environment. CWR22Rv-1 cells stably transfected with oncoprotein *δ*-catenin or GFP vector alone (Zeng *et al*, 2009) were also grown in RPMI1640 medium with 0.25% Gentamycin (G418) (Gibco).

### MTT assay

All cells were treated with Cuc IIa at 1, 10, and 100 *μ*g ml^−1^ for 48 h. The cells were then incubated in MTT (3-(4,5-dimethylthiazol-2-yl)-2,5-diphenyltetrazolium bromide) (ACROS, Morris Plains, NJ, USA). DMSO was applied to lyse the cells and release the formazan purple crystal product. The amount of crystals was read at an absorbance of 562 nm by a microplate reader (Synergy HT, Bio-Tek, Winooski, VT, USA).

### Tumour growth

Mouse hepatocellular carcinoma cell line H22 and Lewis lung carcinoma cells were inoculated subcutaneously into C57 mice with or without Cuc IIa treatments. Cuc IIa was formulated in 2-HP-*β*-CD (2-hydroxypropyl-cycloclextrin) to facilitate solubilisation. 2-HP-*β*-CD is indicated to be more toxicologically benign (Gould and Scott, 2005). It has been shown to be well tolerated in humans so it was adopted for our *in vivo* studies. Cuc IIa in different doses was administered either by oral feeding, peritoneal or intravenous injection. Tumour growth was monitored and their weight was determined at the end of experiments. The mice were maintained according to the Institutional Animal Use Protocol.

### Time-lapse imaging

NIH 3T3 cells were transfected with EGFP-actin (Ballestrem *et al*, 2000) using the transfection reagent Fugene (Roche, Indianapolis, IN, USA). The coverslip with transfected cells was placed onto the stage of Zeiss Axiovert inverted fluorescent microscope (Zeiss, Thornwood, NY, USA) and treated with 10 *μ*g ml^−1^ Cuc IIa. Images of actin clustering in the cells were recorded using time-lapse mode through Hamamatsu-Orca-1 digital camera under the microscope. The camera was set up to record a picture of the cell every 5 min for 4 h using MetaMorph (4.6) software (Molecular Devices, Sunnyvale, CA, USA).

### Immunofluorescent light microscopy

PC-3, CWR22Rv-1, and NCI-H1299 cells were fixed in warm 4% paraformaldehyde. Following permeabilisation in 0.2% Triton X-100, the cells were stained using mouse monoclonal anti-*α*-tubulin DM1*α*, rabbit polyclonal anti-STAT3, or rabbit anti-phosphorylated RhoA (Sauzeau *et al*, 2000a, 2000b). The cells were then incubated with the appropriate Cy3-conjugated secondary antibodies. The nuclei of the cells were stained with Hoechst 33258. The filamentous actin of the cells was stained using rhodamine phalloidin (Molecular Probes, Invitrogen, Carlsbad, CA, USA). The coverslips were mounted on slides using Anti-Fade medium (Invitrogen, Carlsbad, CA, USA) and photographed under the Zeiss Axiovert inverted fluorescent microscope.

### Flow cytometry

To determine cell cycle progression, cultured cells were grown to 60% confluency and treated with Cuc IIa at 10 *μ*g ml^−1^ for 16 h. The cells were stained with propidium iodine (PI) before they were taken for flow cytometry analysis. The DNA content of PI-stained cells was analysed on a FACScan (BD Biosciences, Palo Alto, CA, USA) at 488 nm excitation and emission detected using a 585 nm band pass filter. The percentage of cells in each phase of cell cycle (SubG1, G1, S, and G2/M) was determined using a ModFit 3.1 computer program (Varity Software House, Topsham, ME, USA). The results were analysed by taking the mean and standard error results for each phase.

### Western blot with ECL detection

Cells were treated with Cuc IIa at 1 and 50 *μ*g ml^−1^ for 16 h and lysed in RIPA buffer (1% Triton X-100, 0.5% deoxycholic acid, 0.2% SDS, 150 mM sodium chloride, 2 mM EDTA) with protease inhibitor cocktail (Roche) and pepstatin A. After removing cell debris by centrifugation, protein concentration was determined using BCA method. Some cell lysates were immunoprecipitated using anti-RhoA conjugated sepharose beads and then western blotted with anti-phosphorylated RhoA (serine 188).

The proteins separated by SDS–PAGE were transferred to the nitrocellulose membrane (Optitran, Whatman GmbH, Dassel, Germany) for western blot analyses. The primary antibodies used were against the following antigens: cleaved PARP, PARP, survivin, Histone H3, STAT3, JAK2, phosphorylated STAT3, phosphorylated JAK2, phospho-Histone H3, and phosphorylated RhoA, actin, and GAPDH. After incubation in appropriate secondary antibodies, the membranes were developed with ECL detection reagents. Protein amount were semi-quantified in triplicates using Quantity One (Bio-Rad, Hercules, CA, USA). Statistical analyses were performed and the *P*-values were assigned with the confidence levels set at 95%.

### Detection of DNA ladder with Cuc IIa treatment

PC-3 and CWR22Rv-1 cells were grown to 80% confluence and treated with Cuc IIa at 5 and 40 *μ*g ml^−1^ for 72 h. The internucleosomal DNA was extracted using Apoptotic DNA Ladder Extraction Kit (Biovision, Mountain View, CA, USA). The extracted DNA was run on a 1.2% agarose gel and visualised using ChemiDoc XRS (Bio-Rad).

## Results

### Cuc IIa suppresses cancer cell growth and reduces tumour size

**Figure 1:**
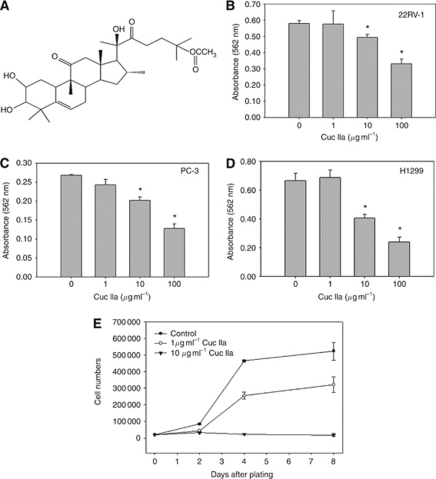
Cuc IIa suppresses cancer cell expansion. (**A**) Chemical structure of Cuc IIa. (**B**) Dose-dependent suppression of prostate cancer CWR22Rv-1 cell expansion measured by MTT assay. (**C**) Dose-dependent suppression of prostate cancer PC-3 cell expansion measured by MTT assay. (**D**) Dose-dependent suppression of lung cancer NCI-H1299 cell expansion measured by MTT assay. The lower absorbance indicates greater cell death. (**E**) Cell growth in CWR22Rv-1 with or without Cuc IIa treatments. CWR22Rv-1 cells were treated with 1 or 10 *μ*g ml^−1^ Cuc IIa and the cell numbers were counted at day 2, 4, and 8. ^*^P<0.05.

We isolated Cuc IIa (Figure 1A) from the roots of *H. amalils* Diels and determined its purity to around 98% (Zhang *et al*, 2006). MTT assays were employed during the initial analyses to determine the effects of Cuc IIa on cancer cell viability. In prostate cancer CWR22Rv-1 cells, 100 *μ*g ml^−1^ Cuc IIa resulted in 43% cell death (Figure 1B). The prostate cancer PC-3 cells showed similar response to Cuc IIa as CWR22Rv-1, with 52% of the cells killed (Figure 1C). Human lung cancer NCI-H1299 cells showed the greatest response to Cuc IIa with 63.9% of cells died (Figure 1D). We thus concluded that Cuc IIa reduced cancer cell viability.

We then applied 1 and 10 *μ*g ml^−1^ Cuc IIa to CWR22Rv-1 cells to examine cell growth in culture (Figure 1E). Compared with control cells, CWR22Rv-1 cells treated with 1 *μ*g ml^−1^ Cuc IIa showed significantly decreased cell numbers by day 2, 4, and 8 following initial cell plating. CWR22Rv-1 cells treated with 10 *μ*g ml^−1^ Cuc IIa showed the reduced growth by day 2 and completely blocked cell expansion by day 4 and beyond (Figure 1E).

To determine if Cuc IIa is effective in reducing tumour growth *in vivo*, we performed either oral feeding, peritoneal or intravenous injection of murine transplanted hepatoma H22 cells in mice. We found that peritoneal injection of Cuc IIa reduced tumour size in a dose-dependent manner (Table 1) while oral administration displayed more moderate results (Table 2). Intravenous injection resulted in a decreased dosing required to suppress the tumour growth indicating higher inhibition efficiency (Table 3). We also tested the effectiveness of Cuc IIa against the growth of LLC model, and the results were quite similar in that it reduced the tumour size in a dose-dependent manner (see Supplementary Table SI). Therefore, Cuc IIa suppressed cancer cell growth and tumour development.

### Cuc IIa disrupts actin cytoskeleton and induces non-reversible aggregation of F-actin

**Figure 2:**
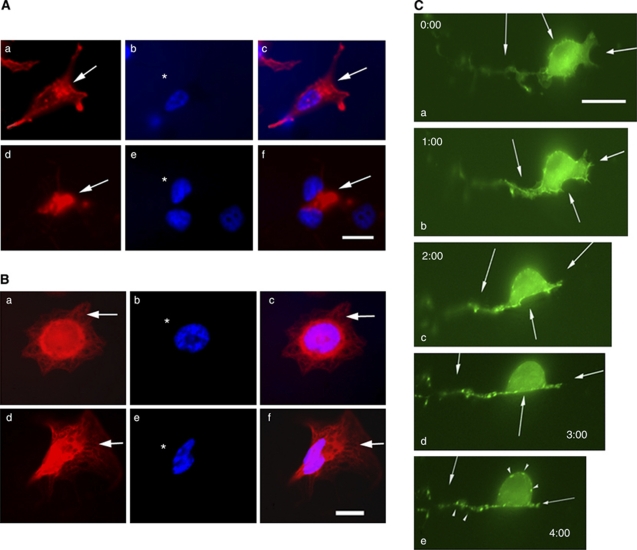
Cuc IIa alters actin cytoskeleton but not microtubule organisation. (**A**) Cuc IIa induces a dramatic actin clustering in cultured CWR22Rv-1 cells. (a–c) Cells without Cuc IIa treatment. (d–f) Cells treated with 50 *μ*g ml^−1^ Cuc IIa. Arrows indicate rhodamine phalloidin staining of F-actin. Bar: 20 *μ*m. (**B**) Cuc IIa does not induce changes in microtubule organisation. (a–c) Cells without Cuc IIa treatment. (d–f) Cells treated with 50 *μ*g ml^−1^ Cuc IIa. (**C**) Time-lapse imaging of NIH3T3 cells transfected with EGFP-actin shows gradual aggregation of actin cytoskeleton and the alteration is not reversible. The cells were grown to 60% confluency and were then transfected with EGFP-actin. The cells were treated with 50 *μ*g ml^−1^ Cuc IIa over a 2-h period followed by a 2-h recovery. Time-lapse images were taken every 5 min. After 2 h of Cuc IIa treatment, the drug was removed and cells were rinsed. The cells were then recorded for an additional 2 h to assess the recovery of actin distribution. Arrows indicate points of increasing actin aggregation with time. Bar: 15 *μ*m.

To investigate the molecular and cellular mechanisms of Cuc IIa effects on suppression of cancer cell expansion, we evaluated cell morphology using immunofluorescent light microscopy. Rhodamine phalloidin staining of CWR22Rv-1 prostate cancer cell lines showed that, unlike control cells in which F-actin was distributed in all of the cytoplasmic compartments (Figure 2Aa and Ac), cells treated with 50 *μ*g ml^−1^ Cuc IIa for 48 h showed severe clustering of F-actin (Figure 2Ad and Af). The microtubules, as demonstrated by the anti-tubulin staining, did not show significant changes (Figure 2B, compare Ba and Bc with Bd and Bf). Therefore, Cuc IIa, like other Cuc family members, targets actin, but not microtubule cytoskeleton.

We further examined the dynamics of F-actin structure treated with Cuc IIa in NIH 3T3 cells that were transfected with EGFP-actin. Transfected cells were recorded using time-lapse imaging under the inverted fluorescent microscope for a period of 4 h while the program MetaMorph took pictures every 5 min (Figure 2C). The cells treated with 50 *μ*g ml^−1^ Cuc IIa showed increasing F-actin aggregation with time (Figure 2C, arrows). After the cells had been treated with Cuc IIa for 2 h, the cells were washed with regular DMEM to rinse off Cuc IIa. After the cells were washed, they were incubated for an additional 2 h to determine if the alteration of F-actin can be reversed. Time-lapse recording showed that the F-actin was continuously constricted and clustered at the different sites in the cells (Figure 2C, arrows and arrowheads). Therefore, Cuc IIa-induced aggregation of actin cytoskeleton is irreversible.

### Cuc IIa-induced cell apoptosis involves survivin and PARP pathways

We then performed western blots to determine which molecular pathways were involved in the cell death induced by Cuc IIa. CWR22Rv-1, PC-3, and NCI-H1299 cells treated with Cuc IIa at 1 and 50 *μ*g ml^−1^ were analysed using antibodies against proteins in the cell cycle and apoptosis-related signaling pathways.

**Figure 3:**
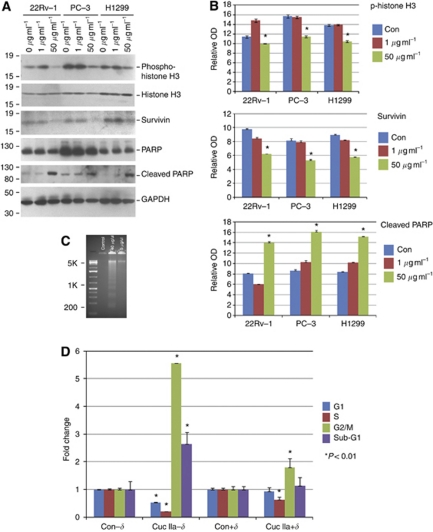
Cuc IIa induces cell cycle alteration and apoptosis involving survivin and PARP. (**A**) Western blots showing Cuc IIa-induced Histone H3 phosphorylation, increased cleavage of PARP, and reduced survivin in prostate cancer CWR22Rv-1 and PC-3 cells as well as lung cancer NCI-H1299 cells. GAPDH is used as loading control. Molecular weight markers are on the left. (**B**) Semi-quantification of Western blots showing Cuc IIa-induced Histone H3 phosphorylation, increased cleavage of PARP, and reduced survivin expression in prostate cancer CWR22Rv-1 and PC-3 cells as well as lung cancer NCI-H1299 cells. ^*^P<0.01. (**C**) Example of DNA fragmentation demonstrated as evidence of apoptosis in PC-3 cells treated with Cuc IIa. In all, 40 *μ*g ml^−1^ Cuc IIa induced DNA fragmentation. (**D**) Flow cytometry analyses demonstrating that the Cuc IIa-induced increases in cell death are suppressed by *δ*-catenin transfection. Con-*δ*: Control Cells without *δ*-catenin overexpression; Con+*δ*: Control Cells with *δ*-catenin overexpression; Cuc IIa-*δ*: Cuc IIa-treated cells without *δ*-catenin overexpression; Cuc IIa+*δ*: Cuc IIa-treated cells with *δ*-catenin overexpression.

Cuc IIa treatments reduced the expression of phospho-Histone H3, consistent with the disruption of cell cycle progression (Figures 3A and B). Inhibition of cell cycle can lead to cell death, and indeed, several apoptosis pathways were activated in Cuc IIa-treated cancer cells. For example, IAP/survivin is a cell cycle related survival factor and an inhibitor of cell death as it blocks caspase-3 and -7 from moving the apoptosis signal further downstream (Li *et al*, 1998; Olie *et al*, 2000). In cells treated with Cuc IIa, the expression of survivin was markedly reduced (Figures 3A and B).

Poly-(ADP-ribose)polymerase is a protein that is critical for cell survival and is also responsible for repairing DNA breaks (Satoh and Lindahl, 1992; Cohen, 1997). When it is cleaved by active cleaved caspase-3, PARP loses its DNA repair function and signals the cell to move into apoptosis (Cohen, 1997). Our western blots showed that, while the expression of total PARP remained unchanged, the level of cleaved PARP was dramatically reduced when the cells were treated with Cuc IIa (Figures 3A and B). Reduction of cleaved PARP suggests that the cell's chromatin and DNA repair machinery was damaged due to Cuc IIa treatments. One effect of caspase/PARP-mediated apoptosis is that the DNA of the cell becomes fragmented and is degraded as the cell dies (Suarez-Huerta *et al*, 2000). Indeed, DNA fragmentation was observed in Cuc IIa-treated PC-3 cells (Figure 3C).

To support the notion that the reduction of survivin expression accounted at least partially for the Cuc IIa effects, we applied Cuc IIa to CWR22Rv-1 cells stably overexpressing *δ*-catenin, an oncoprotein which upregulates survivin expression (Zeng *et al*, 2009) and inhibits actin regulatory protein small GTPase RhoA (Kim *et al*, 2008). We used flow cytometry to document cell cycle progression profiles and cell death. Cells were treated with 10 *μ*g ml^−1^ Cuc IIa for 16 h and stained with PI. Compared with cells without *δ*-catenin overexpression in the absence of Cuc IIa treatment, cells without *δ*-catenin overexpression when treated with Cuc IIa resulted in a 46% reduction of G1 phase, but a decreased S phase to only 20% while having a 5.5-fold increase in G2/M populations, indicating a block after S phase (Figure 3D, Con-*δ* and Cuc IIa-*δ*). The cells treated with Cuc IIa also showed a 2.7-fold increase in sub-G1 phases (Figure 3D, Con-*δ* and Cuc IIa-*δ*). The actin cytoskeleton is critically involved in mitosis and cytokinesis. Failed cytokinesis would show increased G2/M cell numbers. This data is therefore consistent with the disruption of actin cytoskeleton as seen in Figure 2. The 2.7-fold increase in sub-G1 population indicated the increased cell death, probably due to delayed cell cycle progression. Compared with cells with *δ*-catenin overexpression in the absence of Cuc IIa treatment, Cuc IIa treatment in *δ*-catenin overexpressing cells led to a moderate decrease in S phase and a slight increase in G2/M population (Figure 3D, Con+*δ* and Cuc IIa+*δ*). However, there were no significant changes in G1 and sub-G1 population when compared with cells without Cuc IIa treatment (Figure 3D, Con+*δ* and Cuc IIa+*δ*), consistent with the hypothesis that reduced survivin expression is at least a partial mechanism for Cuc IIa-mediated cell death (Figures 3A and B).

### Cuc IIa does not inhibit JAK2 and STAT3 phosphorylation

**Figure 4:**
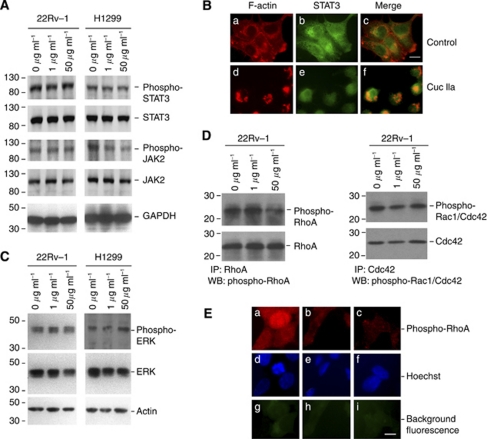
Cuc IIa does not inhibit STAT3/JAK2 phosphorylation but alters RhoA. (**A**) Western blots of STAT3 and JAK2 in prostate cancer and lung cancer cells treated with or without Cuc IIa. Note that there is no significant change in total STAT3 and JAK2 expression and phosphorylation in CWR22Rv-1 cells or NCI-H1299 cells. GAPDH immunoreactivity is used as loading control. Molecular weight markers are on the left. (**B**) Double labeling immunofluorescent light microscopy showing that STAT3 distribution is not changed with or without Cuc IIa. (a–c) Cells without Cuc IIa treatment. (d–f) Cells treated with Cuc IIa. Note: actin in Cuc IIa-treated cells are highly aggregated and clustered. (**C**) Western blots showing that there are no consistent changes in ERK expression and phosphorylation levels in cells treated with or without Cuc IIa. Actin immunoreactivity is used as loading control. Molecular weight makers are indicated on the left. (**D**) Reduced phosphorylation of small GTPase RhoA but not Rac1/Cdc42 in cells treated with Cuc IIa. Left panel: western blots showing that while total RhoA expression is not changed, the serine 188 phosphorylation of RhoA is reduced in CWR22Rv-1 cells treated with 50 *μ*g ml^−1^ Cuc IIa. Right panel: western blots showing that both the total Rac1/Cdc42 expression and their serine 71 phosphorylation are not changed in CWR22Rv-1 cells treated with 50 *μ*g ml^−1^ Cuc IIa. Molecular weight makers are indicated on the left. (**E**) Immunofluorescent light microscopy showing that while control, untreated CWR22Rv-1 cells show a basal serine 188 phosphorylation of RhoA (a, d, and g), Cuc IIa treatments significantly reduce RhoA phosphorylation (b, e, and h indicate cells treated with 1 *μ*g ml^−1^ Cuc IIa but c, f, and i indicate cells treated with 50 *μ*g ml^−1^ Cuc IIa). g, h, and i: background fluorescence.

Recently, some Cuc derivatives have been reported to induce cell apoptosis through JAK2/STAT3 signaling pathway (Kisseleva *et al*, 2002; Sun *et al*, 2005; Thoennissen *et al*, 2009). As survivin is a potential downstream target of JAK2/STAT3 pathway (Scheper *et al*, 2007; Glienke *et al*, 2010), we further explored whether Cuc IIa also inhibited cancer cell expansion by inhibiting JAK2/STAT3 activation. Western blots showed that CWR22Rv-1 and NCI-H1299 cells expressed STAT3 and JAK2 (Figure 4A). However, treatment with Cuc IIa up to 50 *μ*g ml^−1^ did not inhibit STAT3 or JAK2 phosphorylation in CWR22Rv-1 cells, and only marginally reduced JAK2 phosphorylation in NCI-H1299 cells. Therefore, these data suggested that Cuc IIa exerted the cell-death inducing effects downstream of JAK2/STAT3 phosphorylation or expression in these cancer types.

STAT3 activation by JAK2 leads to its nuclear translocation. We therefore, examined whether Cuc IIa changed the STAT3 distribution. NCI-H1299 cells with or without treatment of Cuc IIa were fixed and double labeled with anti-STAT3 and rhodamine phalloidin to label F-actin. Control cells without Cuc IIa treatment showed cytoplasmic and peripheral F-actin staining as expected (Figure 4Ba). STAT3 showed mixed nuclear and cytoplasmic distribution (Figure 4Bb). F-actin only partially co-localised with STAT3 (Figure 4Bc). Cells treated with Cuc IIa showed clustering of F-actin (Figure 4Bd) consistent with above observations in prostate cancer cells (Figure 2A) and NIH 3T3 cells (Figure 2C). While cells treated with Cuc IIa showed remarkable morphological changes, STAT3 still showed a mixed nuclear and cytoplasmic distribution (Figure 4Be and Bf). Additionally, signaling through mitogen-activated protein kinase (MAPK) or extracellular signal-regulated kinase (ERK) is believed to cross talk with JAK2/STAT3 signaling pathway. Western blot showed no consistent activation or inhibition of ERK phosphorylation (Figure 4C). These studies collectively suggested that Cuc IIa-mediated suppression of cancer cell expansion is not dependent on the inhibition of JAK2/STAT3 phosphorylation, expression, or distribution, nor is it dependent on MAPK phosphorylation.

### Cuc IIa induces RhoA phosphorylation

All reported Cuc derivatives display a common targeting feature; that is, they alter actin cytoskeleton organisation (Haritunians *et al*, 2008; Maloney *et al*, 2008). A distinct signaling pathway directly impacting the actin cytoskeletal organisation is the Rho family small GTPases (Lu *et al*, 2009). Rho GTPase pathway also interacts with STAT3 signaling (Debidda *et al*, 2005). Given the phenotypes of cell shape changes and the retraction of cellular protrusions (Figures 2A and C) that are characteristic of RhoA activation, we investigated whether RhoA signaling is altered by Cuc IIa treatments in cancer cells. Western blots showed that the serine 188 phosphorylation of RhoA, which inhibits the RhoGDI interactions with RhoA (Sauzeau *et al*, 2000a, 2000b), was reduced in Cuc IIa-treated CWR22Rv-1 cells (Figure 4D, left panel). The phosphorylation of serine 71 on Rac1/Cdc42, which is an indicator of Rac1/Cdc42 inactivation (Kwon *et al*, 2000), was not changed by Cuc IIa treatment in CWR22Rv-1 cells (Figure 4D, right panel). We confirmed this result by immunofluorescent light microscopy that RhoA phosphorylation at serine 188 was suppressed in CWR22Rv-1 cells (Figure 4E, compare a with c).

## Discussion

Natural occurring medicinal products have made crucial contributions to anti-cancer therapies, such as taxane-based chemo-drugs targeting mitotic microtubule cytoskeletons (Pezzuto, 1997). Originally isolated from the plant *Taxus brevifolia* Nutt, this drug acts on cancer cells by stabilising microtubule cytoskeleton so that the filaments are not able to breakdown (Torres and Horwitz, 1998; Cassinelli *et al*, 2004). As the cell moves through mitosis, the cell is arrested at anaphase (Lanzi *et al*, 2001). The arrested cell most often goes through apoptosis due to mitotic failure; however, some cells can complete mitosis and then undergo programmed cell death (Shi *et al*, 2008).

Cuc is a novel family of anti-cancer drugs from several plants and has received increasing attention in recent years for its apoptosis-inducing effects across multiple cell lines (Wu *et al*, 2002; Yang *et al*, 2007; Thoennissen *et al*, 2009). In our current study, Cuc IIa suppressed cancer cell expansion in cell culture as well as in tumour bearing mouse models, providing a new drug candidate for anti-cancer therapy. Oral feeding had more moderate effects on reducing tumour size, indicating that the first pass through liver may limit effective drug access to the tumours. One of our future researches will focus on improving solubility and bioavailability of Cuc IIa in the *in vivo* studies.

While the overwhelming evidences pointed to the alteration of actin cytoskeletal organisation as the common target, several reports showed that the induction of apoptosis is mediated through JAK2/STAT3 signaling pathway. The present study focused on Cuc IIa purified from the Chinese medicinal plant *H. amalils* and demonstrated, for the first time, that Cuc IIa disrupted actin cytoskeleton and induced apoptosis through novel pathways involving survivin and PARP but independent of JAK2/STAT3 phosphorylation, expression, and distribution.

It has been suggested that the inhibition of JAK2/STAT3 underlies the apoptosis when cancer cells are treated with Cuc. Sun *et al* (2005) showed that Cuc effectively inhibited cancer cell expansion only in STAT3-expressing cells. However, recent study showed that in certain cancer cells without harbouring activated STAT3, K-Ras protected cells from Cuc-induced apoptosis (Escandell *et al*, 2008). It was found that the protective effects can be removed when p53 and p21 were deleted, further supporting the inhibition of mitosis as the initiating event of Cuc-induced apoptosis. Our study is consistent with the observations of Cuc effects on mitosis. We found that even though prostate cancer cells CWR22Rv-1 and lung cancer cells NCI-H1299 displayed JAK2/STAT3 expression and activating phosphorylation, Cuc IIa still induced apoptosis without clearly inhibiting JAK2/STAT3 phosphorylation. Cuc IIa also did not alter nuclear/cytoplasmic distribution of STAT3. We showed that the cell cycle was disrupted with reduced phospho-Histone H3 and survivin. The CWR22Rv-1 cells with overexpression of survivin and inhibition of RhoA protected cancer cells from Cuc IIa to some extent, suggesting that mitotic-related pathways involving survivin (which could be cooperating with p53 and p21 pathway) are potential targets to enhance Cuc IIa's anti-cancer potential. Therefore, not all Cuc family members induce apoptosis by directly inhibiting JAK2/STAT3. Rather, they could target the converging downstream elements such as survivin, indicating a broader benefit of applying Cuc derivatives to attack cancer cells from multiple points.

**Figure 5:**
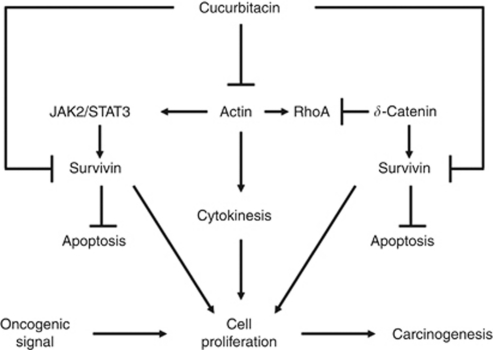
Schematic model illustrating the potential mechanisms of Cuc IIa-induced apoptosis.

One common effect of Cuc derivatives is the disruption of actin cytoskeleton (Duncan *et al*, 1996; Graness *et al*, 2006; Maloney *et al*, 2008; Momma *et al*, 2008). This effect is universal for different Cuc derivatives isolated from a variety of sources (e.g., Cuc B, Cuc I, and Cuc E). The different roles of actin in cell division (cytokinesis) from microtubules (chromosomal movement) underscore the importance of identification of Cuc as novel anti-cancer drugs targeting actin in supporting the established anti-cancer drug taxol targeting microtubules. We thus hypothesise that Cuc IIa arrests cell cycle progression and results in cell death in the following working model (Figure 5). Cuc family disrupts actin dynamics, which initiates two events of cascades in cancer cells. It can inhibit JAK2/STAT3 signaling pathway directly in some cells harbouring activated STAT3. It can also lead to the downregulation of survivin downstream of JAK2/STAT3 without directly targeting JAK2/STAT3 phosphorylation. The failed cytokinesis activates apoptosis pathways, with reduced phosphorylation level of Histone H3 and increased chromatin damage. The reduced PARP function further impairs the repair of damaged DNA, thus suppresses the cell cycle accelerating the apoptosis in p53 and p21-mediated pathways. Therefore, while multiple signaling pathways may be targeted by Cuc derivatives, we propose that the initiating event is the disruption of actin cytoskeleton and its associated signaling events. Significantly, the establishment of a mitotic microtubule modulator such as paclitaxel and actin disrupter such as Cuc IIa will have far reaching implications in future oncologic treatments, as they can potentially suppress two distinct major molecular processes of cancer cell cycle to combat resistances. The further development of Cuc and its improved derivatives may provide unique therapeutic potential to inhibit cancer cell expansion.

## Figures and Tables

**Table 1:** The effects of Cuc IIa on mouse H22 liver cancer through abdominal administration^a^

	**Dosage**	**Administration method**	**Animal number**	**Animal body weight (g)**	**Cancer weight (g)**	**Inhibition**
**Sample**	**(mg kg**^−1^ **per day)**	**Ig × 10qd**	**Start/end**	**Start/end**	**X±s.d.**	**efficiency**
Cuc IIa	90	Ig × 10qd	10/9	21.1/27.6	1.31±0.12	57.05
Cuc IIa	60	Ig × 10qd	10/10	21.0/24.6	1.49±0.12	51.05
Cuc IIa	30	Ig × 10qd	10/10	20.8/25.4	1.95±0.12	36.07
Control	Solvent	Ig × 10qd	20/20	20.8/25.9	3.05±0.28	

a Compared with control, *P*<0.01. The difference is significant.

**Table 2:** The effects of Cuc IIa on mouse H22 liver cancer through pouring into stomach^a^

	**Dosage**	**Administration method**	**Animal number**	**Animal body weight (g)**	**Cancer weight (g)**	**Inhibition**
**Sample**	**(mg kg**^−1^ **per day)**	**Ig × 10qd**	**Start/end**	**Start/end**	**X±s.d.**	**efficiency**
Cuc IIa	90	Ig × 10qd	10/10	19.1/25.7	1.96±0.16	38.17
Cuc IIa	60	Ig × 10qd	10/10	19.3/26.3	2.14±0.20	32.09
Cuc IIa	30	Ig × 10qd	10/10	19.0/26.0	2.31±0.21	27.13
Control	Solvent	Ig × 10qd	10/10	19.3/26.5	3.17±0.32	

a Compared with negative control, *P*<0.01. The difference is significant.

**Table 3:** The effects of Cuc IIa on mouse H22 liver cancer through vein injection^a^

	**Dosage**	**Administration method**	**Animal number**	**Animal body weight (g)**	**Cancer weight (g)**	**Inhibition**
**Sample**	**(mg kg**^−1^ **per day)**	**Ig × 10qd**	**Start/end**	**Start/end**	**X±s.d.**	**efficiency**
Cuc IIa	15	Ig × 10qd	10/10	19.5/25.3	1.48±0.11	55.02
Cuc IIa	10	Ig × 10qd	10/10	19.3/25.9	1.76±0.08	44.12
Cuc IIa	5	Ig × 10qd	10/10	19.4/25.1	2.13±0.20	32.38
Control	Solvent	Ig × 10qd	10/10	19.4/26.0	3.15±0.44	

a Compared with negative control, *P*<0.01. The difference is significant.
