# Cucurbitacin IIa: a novel class of anti-cancer drug inducing non-reversible actin aggregation and inhibiting survivin independent of JAK2/STAT3 phosphorylation

**DOI:** 10.1038/bjc.2012.77

**Published:** 2012-03-13

**Authors:** C Boykin, G Zhang, Y-H Chen, R-W Zhang, X-E Fan, W-M Yang, Q Lu

**Correction to**: *British Journal of Cancer* (2011) **104**, 781–789; doi:10.1038/bjc.2011.10

Since publication of this paper in February 2011, the authors have been made aware of an error in the name of the plant species used in their study. The plant name should be corrected to: *Hemsleya amabilis* Diels (incorrect spelling in paper: Hemsleya amalils Diels).

In addition, the chemical structure of Cuc IIa was incorrectly expressed (shown in Figure 1A in the original publication). Figure 1A is now corrected and shown, below.

## Figures and Tables

**Figure 1:**
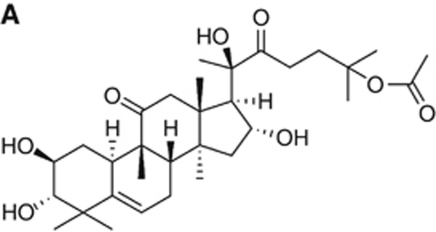
Cuc IIa suppresses cancer cell expansion. (**A**) Chemical structure of Cuc IIa.

