# Supplementary material for: Cucurbitacin IIa: a novel class of anti-cancer drug inducing non-reversible actin aggregation and inhibiting survivin independent of JAK2/STAT3 phosphorylation
Source: Br J Cancer. 2011 Feb 8;104(5):781–9. doi: 10.1038/bjc.2011.10 (PMC3048206; doi:10.1038/bjc.2011.10)
Supplement: Supplementary Table S1 [file bjc201110x1.doc]

Table SI. The effects of Cuc IIa on mouse Lewis lung cancer through vein injection

| Sample | Dosage | Administration method | Animal number | Animal body weight (g) | Cancer weight (g) | Inhibition efficiency |
| --- | --- | --- | --- | --- | --- | --- |
|  | mg/kg/d | Igx10qd | Start/end | Start/end | X±SD |  |
| Cuc IIa | 15 | Igx10qd | 10/10 | 19.5/22.1 | 1.16±0.16 | 59.30 |
| Cuc IIa | 10 | Igx10qd | 10/10 | 19.1/22.9 | 1.50±0.14 | 47.37 |
| Cuc IIa | 5 | Igx10qd | 10/10 | 19.3/22.4 | 1.69±0.11 | 40.76 |
| Control | Solvent | Igx10qd | 20/20 | 18.9/23.0 | 2.85±0.23 |  |

*Compared with control, p<0.01. The difference is significant.
